# Therapeutic Effect of Activated Carbon-Induced Constipation Mice with *Lactobacillus fermentum* Suo on Treatment

**DOI:** 10.3390/ijms151221875

**Published:** 2014-11-28

**Authors:** Huayi Suo, Xin Zhao, Yu Qian, Guijie Li, Zhenhu Liu, Jie Xie, Jian Li

**Affiliations:** 1 College of Food Science, Southwest University, Chongqing 400715, China; E-Mails: birget@swu.edu.cn (H.S.); xiejie1024@swu.edu.cn (J.X.); 2 Department of Biological and Chemical Engineering, Chongqing University of Education, Chongqing 400067, China; E-Mails: zhaoxin@pun.edu (X.Z.); qianyubaby@gmail.com (Y.Q.); quajetlee@gmail.com (G.L.); 3 Science and Technology Administration, Chinese Academy of Agricultural Sciences, Biejing 100081, China; E-Mail: liuzhenhu@caas.cn; 4 Institute of Qinghai-Tibetan Plateau, Southwest University for Nationalities, Chengdu 610041, China

**Keywords:** *Lactobacillus fermentum* Suo, activated carbon, constipation, bisacodyl, gastrointestinal transit

## Abstract

The aim of this study was to investigate the effects of *Lactobacillus fermentum* Suo (LF-Suo) on activated carbon-induced constipation in ICR (Institute of Cancer Research) mice. ICR mice were orally administered with lactic acid bacteria for 9 days. Body weight, diet intake, drinking amount, defecation status, gastrointestinal transit and defecation time, and the serum levels of MTL (motilin), Gas (gastrin), ET (endothelin), SS (somatostatin), AChE (acetylcholinesterase), SP (substance P), VIP (vasoactive intestinal peptide) were used to evaluate the preventive effects of LF-Suo on constipation. Bisacodyl, a laxative drug, was used as a positive control. The normal, control, 100 mg/kg bisacodyl treatment, LB (*Lactobacillus bulgaricus*)-, LF-Suo (L)- and LF-Suo (H)-treated mice showed the time to the first black stool defecation at 90, 218, 117, 180, 155 and 137 min, respectively. By the oral administration of LB-, LF-Suo (L), LF-Suo (H) or bisacodyl (100 mg/kg), the gastrointestinal transit was reduced to 55.2%, 72.3%, 85.5% and 94.6%, respectively, of the transit in normal mice, respectively. In contrast to the control mice, the serum levels of MTL, Gas, ET, AChE, SP and VIP were significantly increased and the serum levels of SS were reduced in the mice treated with LF-Suo (*p* < 0.05). By the RT-PCR (reverse transcription–polymerase chain reaction) and western blot assays, LF-Suo increased the c-Kit, SCF (stem cell factor), GDNF (glial cell line-derived neurotrophic factor) and decreased TRPV1 (transient receptor potential vanilloid 1), NOS (nitric oxide synthase) expressions of small intestine tissue in mice. These results demonstrate that lactic acid bacteria has preventive effects on mouse constipation and LF-Suo demonstrated the best functional activity.

## 1. Introduction

Yak yoghurt is a type of traditional fermentation dairy product of local characteristics in the Qinghai-Tibet Plateau. Yak yoghurt, a nutritious food, not only has a special flavor but also supports anti-oxidization, decreasing cholesterol and enhancing immunity [1]. The rich lactic acid bacterial (LAB) population may contribute to its potential health benefits. LAB contents and qualities of yak yoghurt are influenced by multiple factors, including the living habits of herdsman, yak milk varieties, fermentation temperature, fermentation time, fermentation vessels and so on. Therefore LAB from yak yoghurt is highly different from commercial lactic acid bacteria [2]. Recently, a new LAB was isolated from yak yoghurt from Tibetan habitats and named *Lactobacillus fermentum* Suo.

Constipation is defined medically as fewer than three stools per week and severe constipation as less than one stool per week. It occurs when the colon absorbs too much water [3]. In the current study, activated carbon was orally administered to mice. The GI (gastrointestinal) mucosal surfaces were attached by activated carbon, then the drainage function of GI tract was reduced, these processes caused GI fluid reduction and GI movement slower, the mice constipation model was established by the activated carbon-induced hypofunction of spleen and stomach.

The activated carbon induced constipation mice model was used to demonstrate the effects of drugs for constipation treatment in many studies [4,5]. One study reported that that a megadose of activated carbon can cause digestive tract obstruction [6]. Therefore, in the present study, we examined the functional effects of *Lactobacillus fermentum* Suo in the alimentary tract using an activated carbon-induced constipation mouse model. The GI transit, time of first black stool defecation, histopathological observation and serum levels of motilin (MTL), Gas gastrin, ET (endothelin), SS (somatostatin), AChE (acetylcholinesterase), SP (substance P) and VIP (vasoactive intestinal peptide), which are proteins associated with gastrointestinal mobility, were determined. Bisacodyl was used as a positive control. Bisacodyl is a laxative drug that acts as a stimulant of intestinal peristalsis and acts directly on the colon to produce a bowel movement. It is typically prescribed for the relief of constipation and for the management of neurogenic bowel dysfunction, as well as for bowel preparation prior to medical examinations [7,8,9].

In this study, *Lactobacillus fermentum* Suo (LF-Suo) was used for determining its preventive effect on activated carbon-induced constipation in mice. Firstly, the biological barriers and hydrophobicity of lactic acid bacteria of LF-Suo were examined by *in vitro* tests. Then the anti-constipation effects of LF-Suo were determined by *in vivo* experiments. Further study of its effect on constipation will provide more scientific evidence for the development of better preparations of lactic acid bacteria.

## 2. Results

### 2.1. Biological Barriers and the Ability of Hydrophobicity of Lactic Acid Bacteria

Gastrointestinal survival abilities of lactic acid bacteria were evaluated using man-made gastric juice, bile salt and hydrophobic property tests. LF-Suo showed much higher abilities than LB (Table 1). Especially in different concentrations of bile salt, the growths of LF-Suo were 10–15 times that of LB.

**Table 1:** Resistance to biological barriers and the ability of hydrophobicity for *Lactobacillus fermentum* Suo (LF-Suo).

Stain	Survival in pH 3.0 Manmade Gastric Juice (%)	Hydrophobic Property (%)	Growth in Bile Salt (%)		
			0.3%	0.5%	1.0%
LF-Suo	91.33 ± 5.46	70.31 ± 4.28	27.46 ± 2.23	22.51 ± 1.72	18.22 ± 1.21
LB	27.81 ± 3.41	25.56 ± 2.71	2.61 ± 0.34	1.57 ± 0.37	1.31 ± 0.22

LB: *Lactobacillus bulgaricus*.

### 2.2. Body Weight during the Experiment

**Figure 1:**
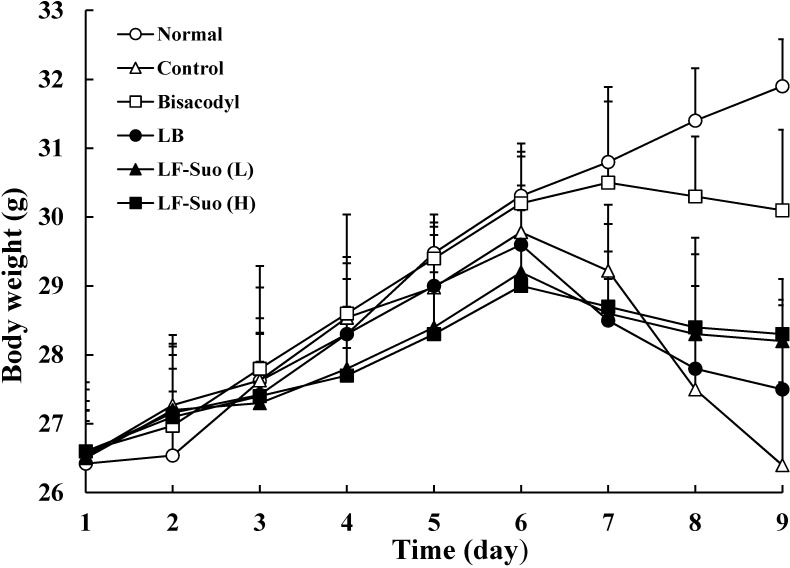
Body weight of mice during the experiment. *n* = 10 ICR (Institute of Cancer Research) mice in each group, bisacodyl: 100 mg/kg b.w. (body weight); LB: *Lactobacillus bulgaricus* (1.0 × 10^9^ CFU/kg b.w.); LF-Suo (L): *Lactobacillus fermentum* Suo (1.5 × 10^9^ CFU/kg b.w.); LF-Suo (H): *Lactobacillus fermentum* Suo (1.0 × 10^9^ CFU/kg b.w.).

Body weight is an important marker for constipation in mice; The body weights of activated carbon-induced constipation mice were lower than normal mice [10]. The drug bisacodyl is a helpful medicine for constipation treatment, it was used for positive control in this study. While the mice were treated with LF-Suo, LB and bisacodyl, the control mice were without treatment for 9 days. The mice were not only continuously treated with LF-Suo, LB and bisacodyl, but also constipation was induced with activated carbon for 3 days. The normal mice were continued without treatment, but were subjected to constipation with lactic acid bacteria. The normal mice had a normal diet and their body weight increased during the experiments. The body weights of the control mice with activated carbon-induced constipation were significantly decreased after six days. As shown in Figure 1, following the initiation of activated carbon-induced constipation, the body weights of the mice in the LF-Suo and LB groups were significantly lower compared with those of the normal mice and the drug treated group of bisacodyl, but higher than activated carbon-induced constipation control mice. LF-Suo treated mice could alleviate the weight loss compared to the LB-treated mice.

### 2.3. Effects of Lactic Acid Bacteria on Diet Uptake and Amount of Water Drinking

Dietary intake of mice in each group increased reposeful from the first to the sixth day. On the afternoon of the sixth day after induction of constipation, dietary intake of control and sample groups were decreased significantly, especially the control group, which decreased the most. But the intake of LF-Suo (H) and LF-Suo (L) were higher than the LB and control group and close to the drug of bisacodyl as shown in Table 2. Drinking water amounts of each mice group was expressed on Table 3, which shows the whole process of the experiment occurs with obvious changes to dietary intake. So, the effect through the observation of the samples on the constipation of mice determined that LF-Suo can relieve dietary intake loss, which means it can relieve anorexia after induced constipation.

**Table 2:** Diet uptake of mice during the experiment.

Treatment	Normal (g)	Control (g)	Bisacodyl (g)	*Lactobacillus bulgaricus* (g)	LF-Suo (×10^9^ CFU/kg b.w.)	
					0.1 (g)	1.0 (g)
1 Day	2.69 ± 0.12	2.66 ± 0.17	2.72 ± 0.14	2.74 ± 0.12	2.64 ± 0.21	2.69 ± 0.16
2 Day	2.73 ± 0.13	2.71 ± 0.21	2.77 ± 0.12	2.75 ± 0.16	2.74 ± 0.17	2.78 ± 0.20
3 Day	2.90 ± 0.11	2.84 ± 0.20	2.86 ± 0.20	2.79 ± 0.14	2.88 ± 0.15	2.93 ± 0.14
4 Day	3.04 ± 0.15	3.01 ± 0.16	3.02 ± 0.15	3.00 ± 0.12	3.09 ± 0.22	3.14 ± 0.12
5 Day	3.08 ± 0.18	3.06 ± 0.15	3.07 ± 0.10	3.12 ± 0.11	3.12 ± 0.15	3.15 ± 0.13
6 Day	3.10 ± 0.16	3.09 ± 0.14	3.12 ± 0.11	3.15 ± 0.16	3.16 ± 0.12	3.16 ± 0.17
7 Day	3.14 ± 0.10	2.60 ± 0.22	2.88 ± 0.22	2.63 ± 0.12	2.71 ± 0.12	2.80 ± 0.10
8 Day	3.15 ± 0.13	2.19 ± 0.15	2.78 ± 0.17	2.40 ± 0.15	2.57 ± 0.18	2.66 ± 0.14
9 Day	3.22 ± 0.13	2.02 ± 0.09	2.70 ± 0.14	2.22 ± 0.13	2.40 ± 0.15	2.48 ± 0.08

*n* = 10 ICR mice in each group, bisacodyl: 100 mg/kg b.w.; *Lactobacillus bulgaricus*: 1.0 × 10^9^ CFU/kg b.w.; LF-Suo: *Lactobacillus fermentum* Suo.

**Table 3:** Drinking amount of mice during the experiment.

Treatment	Normal (mL)	Control (mL)	Bisacodyl Control (mL)	*Lactobacillus bulgaricus* Control (mL)	LF-Suo (×10^9^ CFU/kg b.w.)	
					0.5 (mL)	1.0 (mL)
1 Day	6.24 ± 0.20	6.25 ± 0.20	6.24 ± 0.20	6.24 ± 0.12	6.24 ± 0.20	6.24 ± 0.12
2 Day	6.26 ± 0.21	6.28 ± 0.15	6.30 ± 0.20	6.22 ± 0.16	6.24 ± 0.17	6.26 ± 0.20
3 Day	6.27 ± 0.12	6.24 ± 0.16	6.30 ± 0.16	6.26 ± 0.12	6.26 ± 0.16	6.28 ± 0.17
4 Day	6.25 ± .020	6.25 ± 0.20	6.29 ± 0.15	6.28 ± 0.16	6.28 ± 0.20	6.27 ± 0.21
5 Day	6.32 ± 0.20	6.31 ± 0.15	6.32 ± 0.20	6.30 ± 0.13	6.31 ± 0.27	6.29 ± 0.20
6 Day	6.34 ± 0.20	6.28 ± 0.21	6.34 ± 0.18	6.32 ± 0.10	6.27 ± 0.15	6.28 ± 0.20
7 Day	6.37 ± 0.21	6.14 ± 0.17	6.28 ± 0.15	6.17 ± 0.13	6.25 ± 0.17	6.26 ± 0.15
8 Day	6.38 ± 0.18	5.75 ± 0.18	6.25 ± 0.15	5.93 ± 0.14	6.08 ± 0.16	6.14 ± 0.12
9 Day	6.40 ± 0.22	5.63 ± 0.20	6.21 ± 0.17	5.75 ± 0.12	6.01 ± 0.07	6.12 ± 0.12

*n* = 10 ICR mice in each group, bisacodyl: 100 mg/kg b.w.; *Lactobacillus bulgaricus*: 1.0 × 10^9^ CFU/kg b.w.; LF-Suo: *Lactobacillus fermentum* Suo.

### 2.4. Effect of Lactic Acid Bacteria on Defecation Status of Mice

Constipation refers to bowel movements that are infrequent or hard to pass, characterized by pain in the abdomen and bloating. There are many causes of constipation including medications, poor bowel habits, low fiber diet, abuse of laxatives, hormonal disorders, and disease primarily of other parts of the body that also affect the colon. Therefore defecation is the most important criterion of constipation. This study divided defecation status into three parts: First is defecation weight (g), which can be observed as a main point to estimate constipation situation, if defecation weights are heavy, that means mice have good defecation qualities; Second is particle counts of defecation (pieces), which also clarify the mice constipation situations, more defecation pieces express that mice had good gastrointestinal movement; third is water content of defecation, where higher water content indicates better stool qualities. From the first to the sixth day, defecation weight (g), particle counts of defecation (pieces) and water content of defecation (%) in each group were not significantly different. Defecation weight (g) and particle counts of defecation (pieces) in the bisacodyl group were slightly more than in other groups (Table 4). When it induced constipation, starting from the seventh day to ninth day, defecation weight (g), particle counts of defecation (pieces) and water content of defecation (%) were decreased to 0.37 g, 19 pieces and 16% in the control group. Particle counts of weight and defecation (pieces) were decreased to 0.74 g/38, 0.40 g/21, 0.58 g/27 and 0.67 g/31 pieces, water content of defecation (%) were decreased to 40%, 23%, 29% and 36% in bisacodyl, LB, LF-Suo (L) and LF-Suo (H) groups, respectively. These results demonstrated that LF-Suo has a better defecation status after induced constipation, and thus, that LF-Suo can have a positive effect for constipation relief.

**Table 4:** Defecation status of mice during the experiment.

Treatment	Normal	Control	Bisacodyl	*Lactobacillus bulgaricus*	LF-Suo (×10^9^ CFU/kg b.w.)	
					0.1 (g)	1.0 (g)
1–6 Days (Dose with samples)						
Defecation weight (g)	0.90 ± 0.09	0.94 ± 0.11	1.13 ± 0.07	0.91 ± 0.05	0.92 ± 0.08	0.91 ± 0.05
Particle counts of defecation	35 ± 4	36 ± 7	49 ± 6	36 ± 2	36 ± 5	37 ± 5
Water content of defecation (%)	47 ± 4	47 ± 5	55 ± 5	49 ± 4	45 ± 5	46 ± 6
7–9 Days (High concentration dose with samples and activated carbon)						
Defecation weight (g)	0.91 ± 0.05	0.37 ± 0.06	0.74 ± 0.15	0.40 ± 0.05	0.58 ± 0.04	0.67 ± 0.09
Particle counts of defecation	36 ± 3	19 ± 6	38 ± 5	21 ± 3	27 ± 5	31 ± 4
Water content of defecation (%)	46 ± 5	16 ± 3	40 ± 3	23 ± 2	29 ± 3	36 ± 4

*n* = 10 ICR mice in each group, bisacodyl: 100 mg/kg b.w.; *Lactobacillus bulgaricus*: 1.0 × 10^9^ CFU/kg b.w.; and LF-Suo: *Lactobacillus fermentum* Suo.

### 2.5. Time to the First Black Stool Defecation

**Figure 2:**
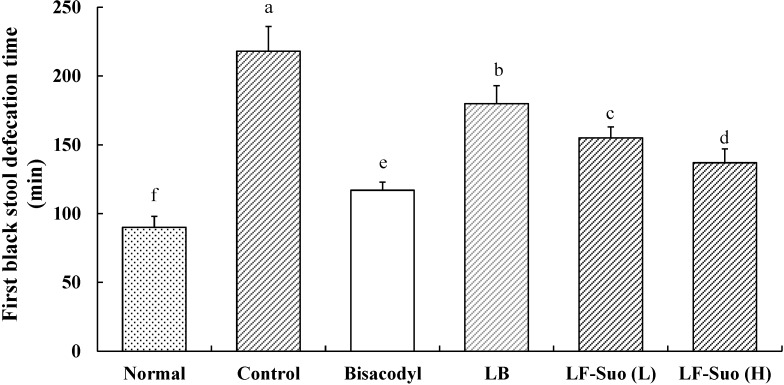
First black stool defecation time of mice after inducing constipation by activated carbon at the last day in the experiment. *n* = 10 ICR mice in each group, bisacodyl: 100 mg/kg b.w.; LB: *Lactobacillus bulgaricus* (1.0 × 10^9^ CFU/kg b.w.); LF-Suo (L): *Lactobacillus fermentum* Suo (1.5 × 10^9^ CFU/kg b.w.); LF-Suo (H): *Lactobacillus fermentum* Suo (1.0 × 10^9^ CFU/kg b.w.); ^a–f^ Mean values with different letters over the bars are significantly different (*p* < 0.05) according to Duncan’s multiple range test.

The time to the first black stool defecation for each group of mice following the administration of activated carbon, which demonstrates the constipation inhibiting effect of different treatments, is shown in Figure 2. The defecation time was the shortest (90 ± 8 min) in the normal group and the longest (218 ± 18 min) in the control group; the defecation time in the bisacodyl group was 117 ± 6 min, higher than that of the normal group. The times to the first black stool defecation for the LB, LF-Suo (L) and LF-Suo (H) groups mice were 180 ± 13, 155 ± 8 and 137 ± 10 min, respectively. According to the defecation time, LF-Suo has the strongest effect on inhibiting constipation.

### 2.6. GI (Gastrointestinal) Transit

**Figure 3:**
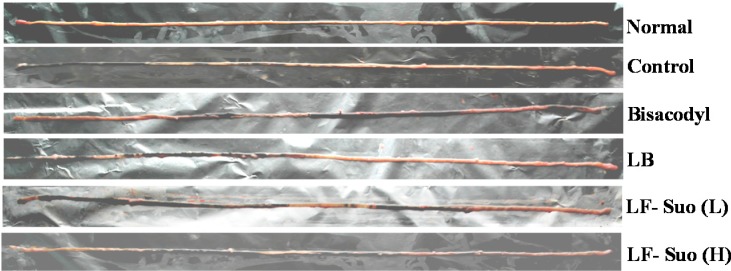
Small intestine of activated carbon-induced constipation model mice. *n* = 10 ICR mice in each group, bisacodyl: 100 mg/kg b.w.; LB: *Lactobacillus bulgaricus* (1.0 × 10^9^ CFU/kg b.w.); LF-Suo (L): *Lactobacillus fermentum* Suo (0.1 × 10^9^ CFU/kg b.w.); LF-Suo (H): *Lactobacillus fermentum* Suo (1.0 × 10^9^ CFU/kg b.w.).

The constipation inhibiting effects of the treatments were determined by GI transit in mice following the administration of activated carbon (0.2 mL/mouse, 10% activated carbon). As shown in the photo of the mice small intestine (Figure 3), LF-Suo helped the activated carbon pass through the small intestine faster than LB and the control did. Moreover, the high dose of LF-Suo increased the transit rate more than the low group. In the bisacodyl-treated group, the mean GI transit was 94.6%, which was higher than that of the control group (42.1; Table 5). The GI transits of the LB, LF-Suo (L) and LF-Suo (H) groups were 55.2% ± 4.6%, 72.3% ± 6.5% and 85.5% ± 6.3%, respectively. LF-Suo increases the GI transit compared with the control, reduces constipation and increases the functional effect.

**Table 5:** Effects of samples on gastrointestinal (GI) transit in activated carbon-induced constipation model mice.

Treatment	Normal	Control	Bisacodyl	*Lactobacillus bulgaricus*	LF-Suo (×10^9^ CFU/kg b.w.)	
					0.1 (g)	1.0 (g)
Small intestine length (cm)	47.9 ± 2.6 ^a^	44.6 ± 4.2 ^a^	47.2 ± 2.2 ^a^	46.4 ± 3.2 ^a^	47.0 ± 3.8 ^a^	47.1 ± 2.5 ^a^
Distance traveled (cm)	47.9 ± 2.6 ^a^	18.8 ± 3.2 ^f^	44.7 ± 1.3 ^b^	25.6 ± 2.5 ^e^	34.0 ± 3.1 ^d^	40.3 ± 1.9 ^c^
GI transit (%)	100.0 ± 0.0 ^a^	42.1 ± 5.6 ^f^	94.6 ± 6.7 ^b^	55.2 ± 4.6 ^e^	72.3 ± 6.5 ^d^	85.5 ± 6.3 ^c^

*n* = 10 ICR mice in each group, bisacodyl: 100 mg/kg b.w.; *Lactobacillus bulgaricus* (1.0 × 10^9^ CFU/kg b.w.); LF-Suo: *Lactobacillus fermentum* Suo; ^a–f^ Mean values with different letters in the same column are significantly different (*p* < 0.05) according to Duncan’s multiple range test.

### 2.7. MTL (Motilin), Gas (Gastrin), ET (Endothelin), SS (Somatostatin), AChE (Acetylcholinesterase), SP (Substance P) and VIP (Vasoactive Intestinal Peptide) Levels in Serum

As shown in Table 6, The MTL, Gas, ET, AchE, SP and VIP levels of normal mice were highest among all the groups while in normal mice the SS level was the lowest. The control mice showed the opposite results, showing the highest level of SS, and the lowest levels of the others. Bisacodyl-treated mice showed close levels of all factors as the normal mice. The MTL, Gas, ET, AchE, SP and VIP levels of LF-Suo treated mice were higher than LB treated mice, however, they were lower than the normal and bisacodyl treated ones. Furthermore, high dose of LF-Suo treated mice showed higher levels of MTL, Gas, ET, AchE, SP and VIP than the low dose treated ones.

**Table 6:** Effect of various samples on serum MTL (motilin), Gas (gastrin), ET (endothelin), SS (somatostatin), AchE (acetylcholine enzyme), SP (substance P) and VIP (vasoactive intestinal peptide) levels in activated carbon-induced constipation model mice.

Level (pg/mL)	Normal	Control	Bisacodyl	*Lactobacillus bulgaricus*	LF-Suo (×10^9^ CFU/kg b.w.)	
					0.5 (g)	1.0 (g)
MTL	173.2 ± 12.6 ^a^	101.3 ± 9.7 ^f^	155.4 ± 8.7 ^b^	119.7 ± 7.7 ^e^	137.5 ± 5.7 ^d^	150.5 ± 6.8 ^c^
Gas	80.2 ± 3.2 ^a^	44.3 ± 2.7 ^f^	73.6 ± 2.6 ^b^	50.2 ± 2.1 ^e^	63.8 ± 2.0 ^d^	70.3 ± 2.3 ^c^
ET	13.9 ± 0.4 ^a^	7.1 ± 0.3 ^f^	12.1 ± 0.3 ^b^	8.4 ± 0.3 ^e^	9.9 ± 0.2 ^d^	10.9 ± 0.2 ^c^
SS	33.2 ± 1.9 ^f^	61.8 ± 1.6 ^a^	40.3 ± 2.0 ^e^	50.2 ± 1.9 ^b^	47.2 ± 1.2 ^c^	43.5 ± 1.3 ^d^
AchE	31.1 ± 1.2 ^a^	12.7 ± 0.9 ^f^	27.8 ± 0.9 ^b^	15.9 ± 0.8 ^e^	22.4 ± 0.9 ^d^	25.6 ± 0.9 ^c^
SP	63.2 ± 2.8 ^a^	37.2 ± 1.9 ^f^	55.3 ± 1.7 ^b^	41.3 ± 0.5 ^e^	47.7 ± 0.8 ^d^	53.1 ± 0.7 ^c^
VIP	52.3 ± 1.9 ^a^	30.6 ± 1.0 ^f^	47.1 ± 1.1 ^b^	33.6 ± 0.9 ^e^	40.3 ± 0.4 ^d^	45.2 ± 0.8 ^c^

*n* = 10 ICR mice in each group, bisacodyl: 100 mg/kg b.w.; *Lactobacillus bulgaricus* (1.0 × 10^9^ CFU/kg b.w.); LF-Suo: *Lactobacillus fermentum* Suo; ^a–f^ Mean values with different letters in the same column are significantly different (*p* < 0.05) according to Duncan’s multiple range test.

### 2.8. Gene Expressions of c-Kit and SCF (Stem Cell Factor)

**Figure 4:**
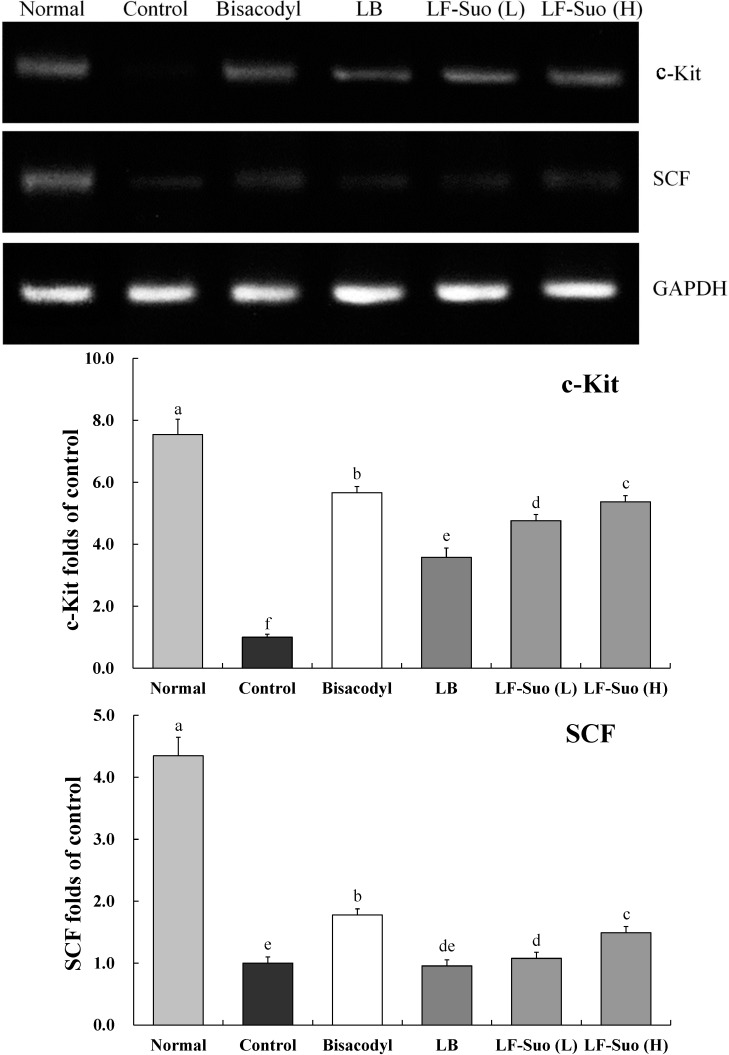
Effects of *Lactobacillus fermentum* Suo on the mRNA expression of c-Kit and SCF in small intestine. *n* = 10 ICR mice in each group, bisacodyl: 100 mg/kg b.w.; LB: *Lactobacillus bulgaricus* (1.0 × 10^9^ CFU/kg b.w.); LF-Suo (L): *Lactobacillus fermentum* Suo (0.1 × 10^9^ CFU/kg b.w.); LF-Suo (H): *Lactobacillus fermentum* Suo (1.0 × 10^9^ CFU/kg b.w.); ^a–f^ Mean values with different letters over the bars are significantly different (*p* < 0.05) according to Duncan’s multiple range test.

**Figure 5:**
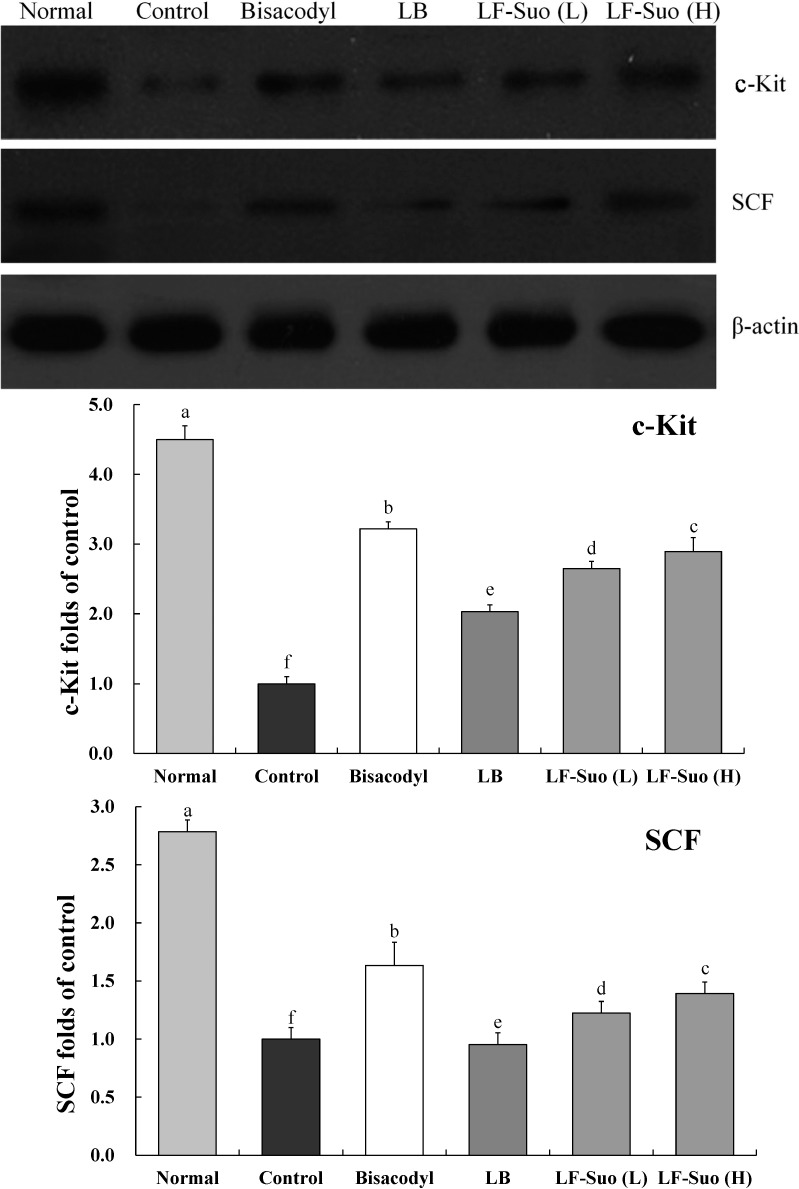
Effects of *Lactobacillus fermentum* Suo on the protein expression of c-Kit and SCF in the small intestine. *n* = 10 ICR mice in each group, bisacodyl: 100 mg/kg b.w.; LB: *Lactobacillus bulgaricus* (1.0 × 10^9^ CFU/kg b.w.); LF-Suo (L): *Lactobacillus fermentum* Suo (0.1 × 10^9^ CFU/kg b.w.); LF-Suo (H): *Lactobacillus fermentum* Suo (1.0 × 10^9^ CFU/kg b.w.); ^a–f^ Mean values with different letters over the bars are significantly different (*p* < 0.05) according to Duncan’s multiple range test.

SCF is a cytokine that binds to the c-Kit receptor, the mRNA and protein expressions of c-Kit and SCF in small intestine tissue were determined by RT-PCR assay after mice treated with LF-Suo. As shown in Figure 4 and Figure 5, treatment with LF-Suo markedly altered the levels of c-Kit and SCF genes. The GAPDH mRNA expression and β-actin protein expression of all groups of mice showed no difference. LF-Suo could increase the expression level of these genes (*p* < 0.05). The higher concentration of LF-Suo showed the more obvious increase.

### 2.9. Gene Expression of TRPV1, GDNF and NOS

**Figure 6:**
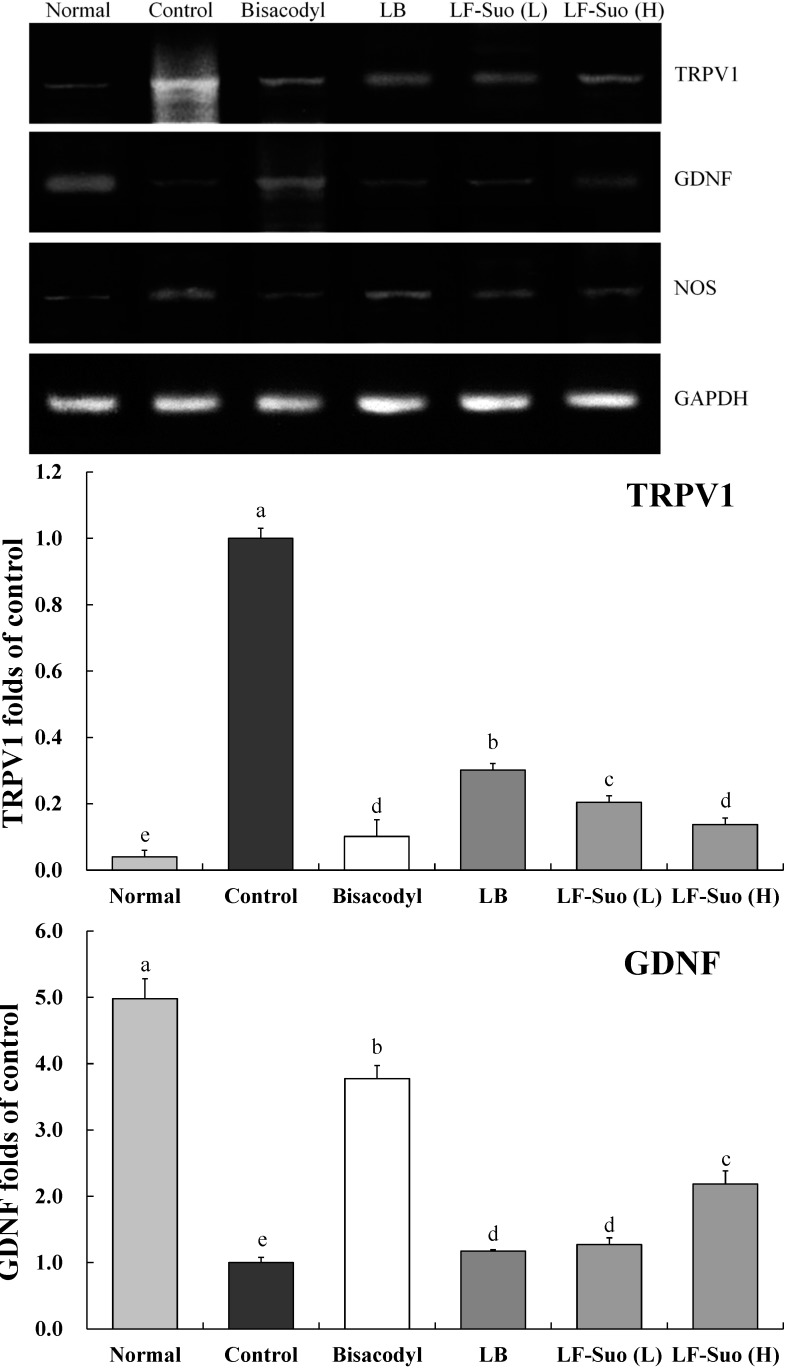

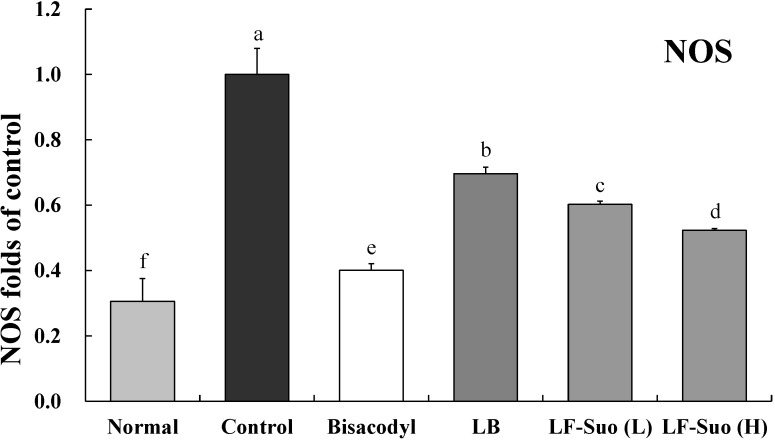
Effects of *Lactobacillus fermentum* Suo on the mRNA expression of TRPV1, GDNF and NOS in small intestine. *n* = 10 ICR mice in each group, bisacodyl: 100 mg/kg b.w.; LB: *Lactobacillus bulgaricus* (1.0 × 10^9^ CFU/kg b.w.); LF-Suo (L): *Lactobacillus fermentum* Suo (0.1 × 10^9^ CFU/kg b.w.); LF- Suo (H): *Lactobacillus fermentum* Suo (1.0 × 10^9^ CFU/kg b.w.); ^a–f^ Mean values with different letters over the bars are significantly different (*p* < 0.05) according to Duncan’s multiple range test.

**Figure 7:**
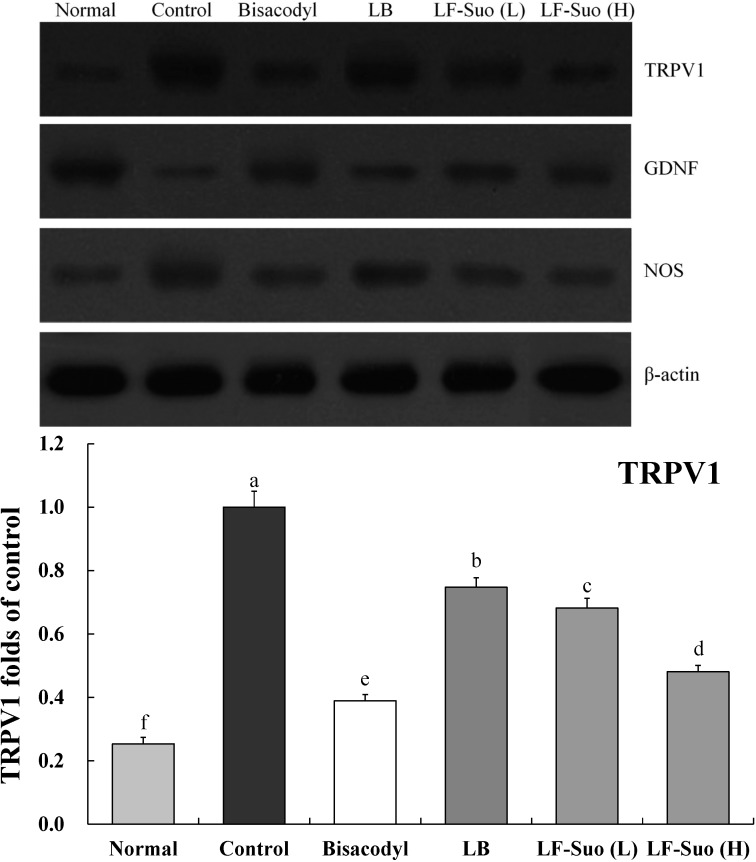

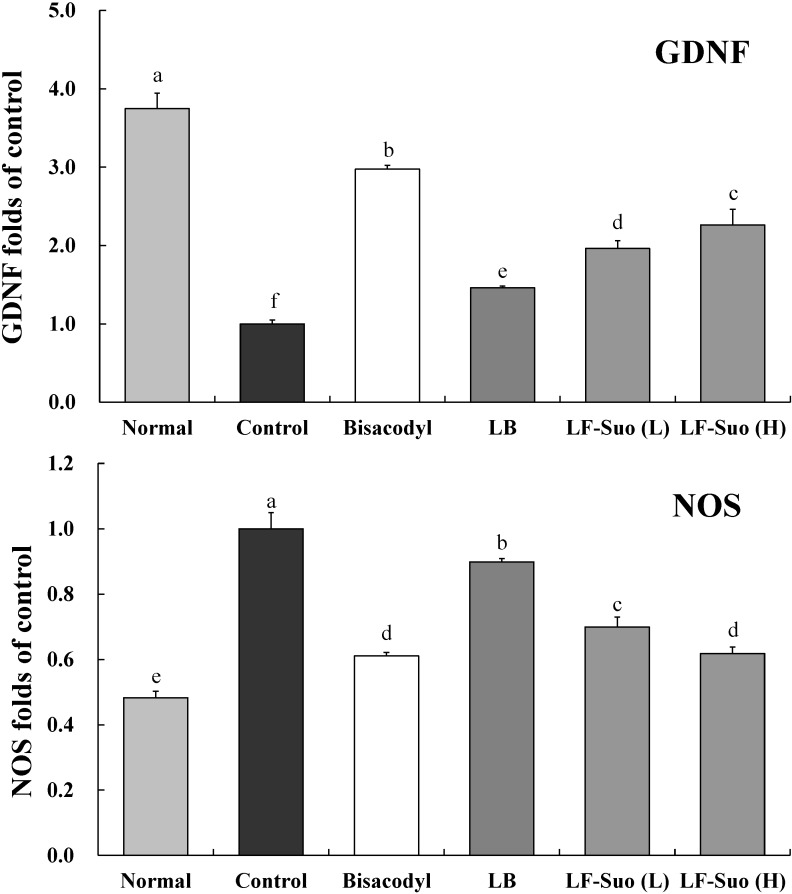
Effects of *Lactobacillus fermentum* Suo on the mRNA expression of TRPV1, GDNF and NOS in small intestine. *n* = 10 ICR mice in each group, bisacodyl: 100 mg/kg b.w.; LB: *Lactobacillus bulgaricus* (1.0 × 10^9^ CFU/kg b.w.); LF-Suo (L): *Lactobacillus fermentum* Suo (0.1 × 10^9^ CFU/kg b.w.); LF-Suo (H): *Lactobacillus fermentum* Suo (1.0 × 10^9^ CFU/kg b.w.); ^a–f^ Mean values with different letters over the bars are significantly different (*p* < 0.05) according to Duncan’s multiple range test.

TRPV1, GDNF and NOS genes are important enteric nerve-related genes. LF-Suo changed the mRNA and protein expression of TRPV1, GDNF and NOS. mRNA expression of GDNF was increased by LF-Suo treatment (*p* < 0.05), TRPV1 and NOS expression showed opposite trends (Figure 6 and Figure 7).

## 3. Discussion

Anorexia is an important symptom in constipation [11]. The observation of dietary and water intake in mice may determine the level of constipation and the inhibitory effects of different substances on constipation. The definition of constipation includes infrequent bowel movements and difficulty during defecation [10,12]. Constipation most commonly occurs when the stool that forms after food is digested moves too slowly (slow transit) as it passes through the digestive tract. Dehydration, changes in diet and activity, and certain drugs are frequently to blame for the slow transit of stools. When stools move slowly, too much water is absorbed from the stool and it becomes hard and dry [13]. Defecation status, dietary intake, water consumption, stool defecation time and GI transit are important standards when investigating constipation.

Probiotics including lactic acid bacteria are usually in food or taken in by oral administration. They have to live through the strong acid conditions in the stomach and upper intestinal tract and reach the destination (usually the large intestine) to colonize and induce physiological effects [14]. Therefore, an external, virtual model of the gastro-intestinal tract was built and the growth potential and trafficability of LF-Suo in the gall bladder and stomach were determined using this model. Its potential probiotic function can be identified by measuring its acid-resistance, cholate tolerance and hydrophobic property [15]. Probiotics increased frequency of bowel movement and decreased constipation. Other reports showed that the probiotic could elevate levels of lactic, acetic and other acids, that could reduce the pH in the intestinal tract, thereby enhancing motility of the intestinal tract and decreasing intestinal tract transit time [16]. LF-Suo showed higher levels of acid-resistance, cholate tolerance and hydrophobic property than *Lactobacillus bulgaricus*, common lactic acid bacteria, and these qualities could help provide positive functional effects of LF-Suo for human.

The surviving thallus passing the stomach would get in touch with the cholate in small intestine. Cholate tolerance of lactic acid bacteria is taken as a standard to identify potential probiotics [17]. Apart from being able to resist cholate in small intestines, lactic acid bacteria are supposed to have great adhesion on mucous membrane of small intestine. Therefore, the hydrophobic property of lactic acid bacteria would be taken as another standard [18]. By imitating stomach and intestines and building external model to test the tolerance ability of probiotics to gastric juice and cholate, we learned that lactic acid bacteria have stronger tolerance ability to gastric juice and cholate than common lactic acid bacteria like *Lactobacillus bulgaricus*. The results showed that LF-Suo might have high survival rate in the stomach and intestines, and thus LF-Suo could increase the functional effects in mice. Meanwhile, the experiment to test the hydrophobic property proves that lactic acid bacteria of LF-Suo entering the small intestine have stronger adhesion than lactic acid bacteria and more of them can colonize in the small intestine to take effect.

Excrement that stays longer in the small intestine and the possibility of harmful bacteria that utilizing excrement as food to reproduce continuously, could threaten intestinal health and compound the negative effects of constipation. Other organs would suffer from damage if intestines absorb harmful substance produced by harmful bacteria [19]. The small intestine would be alkaline after suffering from constipation and lactic acid bacteria would produce large amounts of acid to adjust the pH value, thus making the environment in intestines disadvantageous to the growth of harmful bacteria and even kill them [20]. Lactic acid bacteria can also promote the intestinal tract movement and produce active material beneficial to the intestine, thus preventing constipation.

The serum levels of MTL, Gas, ET, AChE, SP and VIP in patients with constipation are lower than those in healthy individuals, while the SS levels are higher [21,22]. The main function of MTL is to increase the migrating myoelectric complex component of GI motility and stimulate the production of pepsin. It is one of the intestinal hormones responsible for the proper filling and emptying of the GI system in response to intake of food, as well as hunger stimuli and responses [23]. Gas is a polypeptide hormone secreted by certain cells of the pyloric glands, which strongly stimulates the secretion of gastric acid and pepsin, and weakly stimulates the secretion of pancreatic enzymes and gallbladder contraction [24]. Gas produces effects throughout the GI tract, including promoting GI secretion, increasing GI movement and promoting pyloric sphincter relaxation. ET plays an important role in the stability of vascular tension and maintains the basic cardiovascular system. Constipation not only causes disease, including intestinal obstruction and other serious diseases, but it also induces or aggravates cardiocerebrovascular diseases in the elderly [25]. Somatostatin, which is homologous with cortistatin, can suppress the release of gastrointestinal hormones, reduce smooth muscle contractions, and decrease the rate of gastric emptying, all of which leading to constipation [26]. Stools are formed from the non-digestible components of food after water is either absorbed or secreted in the large intestine. Mucus is also produced in the large intestine to provide viscosity. Thin segments of muscle line the intestinal tract and contract and relax in concert to propel the stool forward. Muscle contraction and mucus secretion are regulated by acetylcholine [27]. Patients with slow transit constipation have abnormal neurotransmitters in the muscular layer of their intestinal walls. These abnormalities include a deficiency of a peptide known as SP, which is thought to contribute to peristalsis [28]. Disturbances in the normal neural content of VIP in the bowel wall in idiopathic constipation and diverticular disease may initiate or contribute to the functional changes observed in these disorders [29].

*GAPDH* gene, a housekeeping gene in most cells, is commonly used as a loading control for qPCR [30]. In western blotting assay, β-actin is also used as a loading control for the protein degradation of cancer cells [31]. In this study, no differences were observed in GAPDH and β-actin expression among all groups, which enable their use as controls for other genes. Interstitial cells of Cajal (ICC) is a kind of mesenchymal cell between the intestinal nervous system and smooth muscle, which plays an important role in regulating intestinal nerve signals to smooth muscle cells [32]. According to research, patients with constipation have less ICC in intestines. C-Kit is the specific marker of ICC, which is the key to the proliferation of ICC. Brading *et al.* found that the concentration of SCF is very important to the cultivation of the ICC. Without SCF, the cultivation of ICC cannot survive. Further study on animals with constipation found that with lower amounts of ICC in colon tissue of mice with constipation, c-Kit and SCF expression levels will decline [33]. SCF and c-Kit in mice with constipation are less than normal mice, this difference between constipation mice and normal mice can be reduced by LF-Suo.

TRPV1 has a close relation between bowel movement and absorption. Activated TRPV1 can trigger the releasing of the neurotransmitter, which leads to the disorder of small intestine movement. The increasing of TRPV1 expression is a striking phenomenon of the intestinal damage. Due to the damage of intestines and disorder of movements, constipation patients have a much higher TRPV1 expression [34]. GDNF is a kind of active protein factor which can control the growth and development of nerve cells, protect and repair the damaged nervous fiber. GDNF can adjust ganglion cells to function. Enhancement of GDNF content can contribute to repair the damaged intestines, which can avoid constipation [35]. NOS enzyme is key to produce endogenous NO which widely exists in gastrointestinal tissues from the esophagus to the anus sphincter and plays an important role in adjusting gastrointestinal movement. The increase of NO has striking effects on colonic motility disorder of STC patient. NOS control can reduce the content of NO, which is a feasible way to control constipation [36]. LF-Suo could significantly increase the GDNF expression of small intestinal tissue and reduce the TRPV1 and NOS expression in the process of mice with constipation, which could inhibit constipation.

## 4. Materials and Methods

### 4.1. Microorganism Strains

*Lactobacillus fermentum* Suo was isolated and identified from yak yoghurt of Hongyuan grassland houseland (Hongyuan, Ngawa Prefecture of Sichuan Province, China) and deposited with the China Center for Type Culture Collection (CCTCC, Wuhan, China), bearing CCTCC Accession Number M2013511. *Lactobacillus bulgaricus* was purchased from Institute of Microbiology of the Chinese Academy of Sciences, Beijing, China.

### 4.2. Animals

Seven-week-old female ICR mice (*n* = 120) were purchased from the Experimental Animal Center of Chongqing Medical University (Chongqing, China). The mice were maintained in a temperature- and humidity-controlled (temperature 25 ± 2 °C, relative humidity 50% ± 5%) facility with a 12-h light/dark cycle and free access to a standard rat chow diet and water.

### 4.3. Endurance Capacity of Lactic Acid Bacteria to pH 3.0 Manmade Gastric Juice

The artificial gastric juice was made by 0.2% of NaCl and 0.35% of pepsin, adjusted to pH 3.0 and then vacuum-filtered to remove bacteria in the clean bench. 5 mL of reactivated bacteria culture was centrifuged at 3000 rpm for 10 min, and the bacteria pellet was collected and re-suspended in 5 mL of sterile saline. 1 mL of the suspension was mixed with 9 mL of the artificial gastric juice and incubated in thermostatic oscillator at 37 °C and 300 rpm. A sample volumes of 200 μL was pipetted at 0 and 3 h and plates were poured with de Man, Rogosa and Sharpe (MRS) agar and incubated at 37 °C for 48 h. Colony Forming Units (CFU) was counted and the survival rate was determined [15].

### 4.4. Determination of Bacterial Tolerance of Bile Salt (Oxgall) in Different Concentration

A volume of 100 μL of reactivated bacteria culture was inoculated with 2% (*v*/*v*) MRS-thio (MRS plus 0.2% of sodium thioglycolate (Zibo Gaohuan Fine Chemical Co., Ltd., Zibo, Shandong, China)) broth which contained 0.0% oxgall (the control), 0.3% oxgall, 0.5% oxgall and l.0% oxgall respectively. After incubation at 37 °C for 24 h, each culture was measured for its OD (optical density) value, and the tolerance of bacteria strain to oxgall was determined by comparing the OD of oxgall tube with that of the control tube [15].

### 4.5. Determination the Hydrophobic Property of Lactic Acid Bacteria

A 5 mL sample of reactivated bacteria culture was centrifuged at 3000 rpm for 10 min. The bacterial pellet was collected and re-suspended in 5 mL of phosphate buffer saline (PBS) buffer (50 mM, pH 6.5), and the suspension was centrifuged at 3000 rpm for 10 min; The process was repeated once again. Using PBS buffer as the blank of absorption, the final bacteria suspension was adjusted by PBS buffer to make a 1.00 absorbance (denoted as A_0_) at 560 nm. 4 mL of the adjusted bacteria suspension was added with 0.8 mL of dimethylbenzene, vibrated for 30 s and then placed for stratification. The aqueous layer was measured for the absorbance (denoted as A) at 560 nm (blank: PBS buffer) and the results were recorded [37].

### 4.6. Induction of Constipation in Mice

To investigate the preventive effects of LF-Suo against activated carbon-induced constipation, the animals were divided into six groups with 20 mice in each. The experimental design was as follows: The normal and control groups were fed a normal diet for 9 days and the high concentration LF-Suo, low concentration LF-Suo and LB groups were received 1 × 10^9^, 1 × 10^8^ and 1 × 10^9^ CFU/mL dose oral administration for 2 mL, the drug cure group mice were treated with a 100 mg/kg dose of bisacodyl dissolved in water for 9 days. The control and treatment groups received an oral administration of activated carbon (0.2 mL 10% activated carbon, *w*/*w*; Activated carbon dissolved in 10% arabic gum) at 6 p.m. from the sixth to ninth day to induce constipation [38]. The body weight, dietary intake, water intake, stools weight and stools moisture were determined at 9:00 a.m. everyday.

### 4.7. Measurement Defecation Status of Mice

This measurement was performed to determine the prokinetic action of lactic acid bacteria was capable of propagating a prokinetic signal along the entire length of the gastrointestinal tract. The excreted fecal pellets of individual mice were collected daily at 9:00 a.m. throughout the duration of the experiment. The total number, weight, and water content of the pellets were determined. The water content was calculated as the difference between the wet and dry weight of pellet. After 16 h, the mice of control and sample groups received 10% activated carbon, and the normal group was administered 10% arabic gum through intragastric gavage. Then the animals were immediately placed in small transparent cages individually and allowed access to their diets and tap water *ad libitum*. The length of time from carbon meal administration to the appearance of darkened defecation was recorded. Feces were collected, counted, water content were measured and weighted after intragastric gavage administration.

### 4.8. GI Transit and Defecation Time

Mice were fasted for sixteen hours from the ninth day at 6 p.m.; However, they were not deprived of water. After 16 h, the mice in the control and treatment groups received an oral administration of 10% activated carbon while the mice in the normal group received an oral administration of 10% arabic gum. Thirty minutes later, mice were sacrificed by cervical dislocation under anesthesia with diethyl ether. Ten mice in each group were dissected and the small intestine from the pylorus to the blind intestine was carefully removed. The GI transit of each mouse was calculated as the percentage of the distance traveled by the activated carbon meal relative to the total length of the small intestine. The following equation was used to calculate GI transit: GI transit (%) = distance traveled by the activated carbon/total length of the small intestine × 100%. The remaining 10 mice of each group were used to measure the time to the first black stool defecation following the oral administration of 10% activated carbon.

### 4.9. MTL, Gas, ET, SS, AChE, SP and VIP Levels in Serum

MTL, Gas, ET, SS, AChE, SP and VIP levels in the serum were determined using radioimmunoassay kits (Beijing Puer Weiye Biotechnology Co., Ltd., Beijing, China). The serums of mice were collected from heart following surgery.

### 4.10. RT-PCR Assay

Total RNA in tissues were extracted via Trizol (Invitrogen, Carlsbad, CA, USA) according to the instructions. The total RNA concentration from each sample group was adjusted to the same level after testing its purity with ultraviolet radiation. The same amount of RNA (2 μg) was taken from the samples, then 1 μL oligodT18, RNase, dNTP and 5× buffer 10 μL enzyme MLV were added respectively. In 10 μL body fluid, cDNA was synthesized at 37 °C for 120 min and 99 °C for 4 min and 4 °C for 3 min respectively. The target genes were then reversely transcribed and amplified (Table 1). The reaction conditions were initial denaturation for 5 min at 95 °C, anneal 50 s at 58 °C, extend for 90 s at 72 °C, then repeating it for 40 times, extend for 10 min at 95 °C. In the end, the expressions of the final products were determined with 2% agarose gel electrophoresis [39].

### 4.11. Western Blot Assay

Protein lysates were added into the tissues after rinsing with pre-cooled PBS for 3 times, lysed at 4 °C and centrifuged (10,000 r/min) for 15 min. Supernatant proteins were then extracted and mixed with sodium dodecyl-sulfate-polyacrylamide gelelectrophoresis (SDS-PAGE) loading buffer. Primary antibodies were put into them after SDS-PAGE gel electrophoresis and transfer to a membrane, and the proteins were placed overnight at 4 °C. Then horseradish peroxidase-conjugated secondary antibodies were incubated with the proteins at room temperature. In the end, immunoreactive proteins were tested with a chemiluminescent enhanced chemiluminescence assay kit (GE Healthcare, Uppsala, Sweden) and observed with a LAS3000 luminescent image analyzer (Fujifilm, Tokyo, Japan) with β-actin as internal reference [39].

### 4.12. Statistical Analysis

Data are presented as the mean ± SD (Standard Deviation). Differences between the mean values for individual groups were assessed with a one-way ANOVA with Duncan’s multiple range test. Differences were considered significant when *p* < 0.05. SAS version 9.1 (SAS Institute Inc., Cary, NC, USA) was used for statistical analyses.

## 5. Conclusions

The aim of the current study was to investigate whether *Lactobacillus fermentum* Suo has a preventive effect against activated carbon-induced constipation in mice. *Lactobacillus fermentum* Suo has the better qualities of acid-resistance, cholate tolerance and hydrophobic property than *Lactobacillus bulgaricus*. In the mice treated with *Lactobacillus fermentum* Suo, the results demonstrated that the time to the first black stool defecation was only a little longer than that in mice treated with bisacodyl. The GI transit was longer than that in the control mice and was similar to that in the bisacodyl group. Various serum levels, including MTL, Gas, ET, AChE, SP and VIP in the *Lactobacillus fermentum* Suo-treated mice were higher compared with those in the control mice and common *Lactobacillus bulgaricus* treated mice, and the SS levels demonstrated the opposite tendency. *Lactobacillus fermentum* Suo-treated mice showed higher mRNA and protein expression levels of c-Kit, SCF, GDNF, and lower levels of TRPV1, NOS than those in *Lactobacillus bulgaricus* treated mice and control mice. These results suggest that *Lactobacillus fermentum* Suo has a significant preventive effect on activated carbon-induced constipation in mice.
